# (2*S*,4a*R*,3*S*,8a*R*,9*R*,10*R*)-1,4-Diallyl-2,3-diphenyl­perhydro­quinoxaline

**DOI:** 10.1107/S1600536808011276

**Published:** 2008-04-26

**Authors:** Fang Chen, Heng-Yun Ye

**Affiliations:** a Ordered Matter Science Research Center, College of Chemistry and Chemical Engineering, Southeast University, Nanjing 210096, People’s Republic of China.

## Abstract

In the title compound, C_26_H_32_N_2_, the cyclo­hexane and piperazine rings each adopt a chair conformation. Both phenyl rings and the two propen-3-yl residues are in equatorial positions. There are no C—H⋯N hydrogen bonds nor π–π inter­actions between the aromatic rings. The absolute configuration was assigned with reference to the starting material.

## Related literature

For an olefin–copper (I) complex with high anisotropy, see: Ye *et al.* (2007 ▶). For examples of the structure of olefins, see: Bond & Davies (2001 ▶); Presenti *et al.* (2001 ▶); Wang & Ye (2008 ▶).
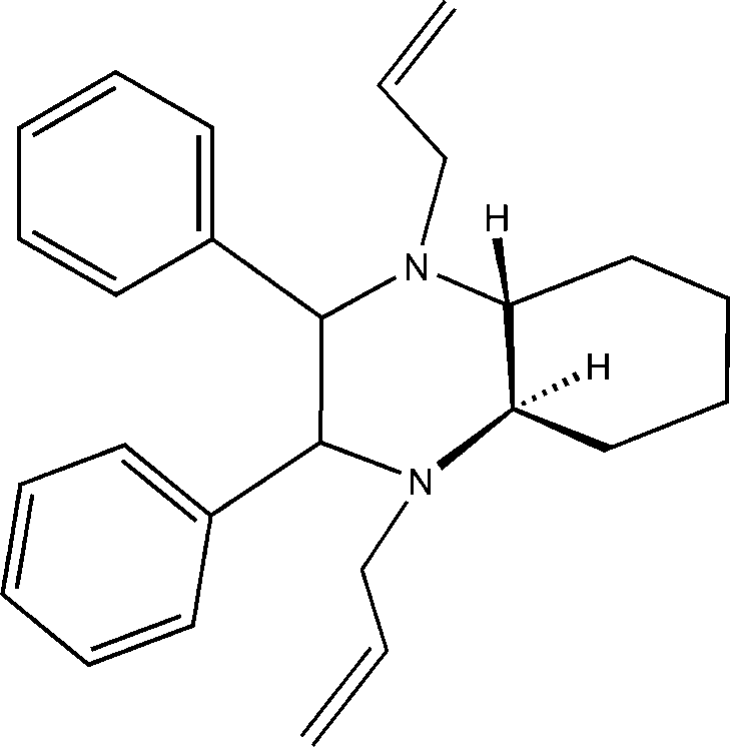


## Experimental

### 

#### Crystal data

C_26_H_32_N_2_
*M* *_r_* = 372.54
Orthorhombic, 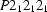

*a* = 6.509 (4) Å
*b* = 17.437 (10) Å
*c* = 19.757 (12) Å
*V* = 2242 (2) Å^3^
*Z* = 4
Mo *K*α radiation
μ = 0.06 mm^−1^
*T* = 293 (2) K
0.35 × 0.15 × 0.15 mm

#### Data collection

Rigaku SCXmini diffractometer
Absorption correction: multi-scan (*CrystalClear*; Rigaku, 2005 ▶) *T*_min_ = 0.808, *T*_max_ = 1.000 (expected range = 0.801–0.990)
22256 measured reflections
2923 independent reflections
2452 reflections with *I* > 2σ(*I*)
*R*_int_ = 0.040

#### Refinement

*R*[*F* ^2^ > 2σ(*F* ^2^)] = 0.059
*wR*(*F* ^2^) = 0.154
*S* = 1.13
2923 reflections
254 parameters
H-atom parameters constrained
Δρ_max_ = 0.15 e Å^−3^
Δρ_min_ = −0.13 e Å^−3^

### 

Data collection: *CrystalClear* (Rigaku, 2005 ▶); cell refinement: *CrystalClear*; data reduction: *CrystalClear*; program(s) used to solve structure: *SHELXS97* (Sheldrick, 2008 ▶); program(s) used to refine structure: *SHELXL97* (Sheldrick, 2008 ▶); molecular graphics: *SHELXTL* (Sheldrick, 2008 ▶); software used to prepare material for publication: *SHELXTL*.

## Supplementary Material

Crystal structure: contains datablocks I, global. DOI: 10.1107/S1600536808011276/bt2698sup1.cif

Structure factors: contains datablocks I. DOI: 10.1107/S1600536808011276/bt2698Isup2.hkl

Additional supplementary materials:  crystallographic information; 3D view; checkCIF report

## Figures and Tables

**Fig. 1.**
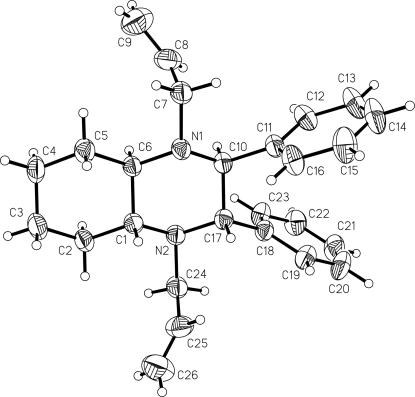
Molecular conformation of the title compound with the atomic numbering scheme and displacement ellipsoids drawn at the 30% probability level.

**Fig. 2.**
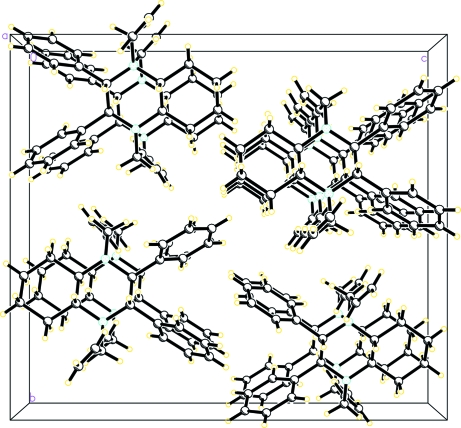
The crystal packing of the title compound viewed along the a axis.

## supplementary crystallographic information

### Comment

Recently, we have reported large anisotropy of an olefin copper (I) complex (Ye, *et al.*, 2007). As a part of our ongoing investigations in this field we have determined the crystal structure of the title compound  (Fig. 1).

The distances of the C=C double bonds [C8-C9 1.284 (5)Å, C25-C26 1.235 (5)Å] are slightly shorter than those found in other olefin compounds (Bond  *et al.*, 2001; Presenti  *et al.*, 2001). This might be due to an increased thermal vibration of the terminal   C atoms. The two phenyl and the two propen-3-yl residues are located in an equatorial postion. The cyclohexane ring  and the piperazine ring adopt a chair conformation. The two aromatic rings are gauche to each other [torsion angle C11—C10—C17—C18  -58.0 (2)°]. The dihedral angle  between the two aromatic rings is 50.66 (0.10)°.

### Experimental

(4aR,8aR)-2,3-Diphenyl-4a,5,6,7,8,8a-hexahydroquinoxaline (Wang *et al.*(2008)  (2.0 g, 6.9 mmol) was dissolved in methanol (30 ml) and NaBH_4_ (0.3 g) was added to the solution portionally. The mixture was stirred at room temperature for 3 h. The resulting solution was poured into ice water (200 mL), then extracted with dichlomethane (30 ml × 2). The organic phase was washed with saturated sodium chloride aqueous solution (20 mL) then dried with anhydrous sodium sulfate. After removing the solvent, the residue, potassium carbonate (3 g) and ethanol (20 mL) were placed to a 50 mL round bottom flask. After stirred for 15 min, a solution of allyl bromide (1.4 g, 11.5 mmol) in ethanol (10 mL) was added to the reaction mixture. The mixture was heated to reflux for *ca* 2 h until the starting material disappeared with TLC detection. The resulting solution was cooled and filtered off. The solvent was removed under reduced pressure to give a white semisolid product. The crude product was recrystallized by slowly evaporating an acetone solution to yield colorless block-like crystals.

### Refinement

All H atoms were found in a difference electron-density map. Nevertheless, they were placed at idealized positons and refined using a riding model with C_methine_—H = 0.98Å, C_methylene_—H = 0.97Å, C_aryl_—H =0.93Å, C_ethylene_—H =0.93Å, and *U*_iso_H = 1.2 *U*_eq_C. Due to the absence of significant anomalous scattering effects, 2187 Friedel pairs were merged. The absolute configuration was set according the starting material.

### Crystal data

**Table:**

C_26_H_32_N_2_	*F*_000_ = 808
*M**_r_* = 372.54	*D*_x_ = 1.101 Mg m^−^^3^
Orthorhombic, *P*2_1_2_1_2_1_	Mo *K*α radiation λ = 0.71073 Å
Hall symbol: P 2ac 2ab	Cell parameters from 15922 reflections
*a* = 6.509 (4) Å	θ = 3.3–27.5º
*b* = 17.437 (10) Å	µ = 0.06 mm^−^^1^
*c* = 19.757 (12) Å	*T* = 293 (2) K
*V* = 2242 (2) Å^3^	Block, colorless
*Z* = 4	0.35 × 0.15 × 0.15 mm

### Data collection

**Table:**

Rigaku SCXmini diffractometer	2923 independent reflections
Radiation source: fine-focus sealed tube	2452 reflections with *I* > 2σ(*I*)
Monochromator: graphite	*R*_int_ = 0.040
Detector resolution: 13.6612 pixels mm^-1^	θ_max_ = 27.4º
*T* = 293(2) K	θ_min_ = 3.3º
ω scans	*h* = −8→8
Absorption correction: multi-scan(CrystalClear; Rigaku, 2005)	*k* = −22→22
*T*_min_ = 0.809, *T*_max_ = 1.000	*l* = −25→25
22256 measured reflections	

### Refinement

**Table:**

Refinement on *F*^2^	Secondary atom site location: difference Fourier map
Least-squares matrix: full	Hydrogen site location: inferred from neighbouring sites
*R*[*F*^2^ > 2σ(*F*^2^)] = 0.059	H-atom parameters constrained
*wR*(*F*^2^) = 0.154	*w* = 1/[σ^2^(*F*_o_^2^) + (0.0674*P*)^2^ + 0.2312*P*] where *P* = (*F*_o_^2^ + 2*F*_c_^2^)/3
*S* = 1.13	(Δ/σ)_max_ < 0.001
2923 reflections	Δρ_max_ = 0.15 e Å^−^^3^
254 parameters	Δρ_min_ = −0.13 e Å^−^^3^
Primary atom site location: structure-invariant direct methods	Extinction correction: none

### Special details

**Table:**

Geometry. All e.s.d.'s (except the e.s.d. in the dihedral angle between two l.s. planes) are estimated using the full covariance matrix. The cell e.s.d.'s are taken into account individually in the estimation of e.s.d.'s in distances, angles and torsion angles; correlations between e.s.d.'s in cell parameters are only used when they are defined by crystal symmetry. An approximate (isotropic) treatment of cell e.s.d.'s is used for estimating e.s.d.'s involving l.s. planes.
Refinement. Refinement of *F*^2^ against ALL reflections. The weighted *R*-factor *wR* and goodness of fit *S* are based on *F*^2^, conventional *R*-factors *R* are based on *F*, with *F* set to zero for negative *F*^2^. The threshold expression of *F*^2^ > σ(*F*^2^) is used only for calculating *R*-factors(gt) *etc*. and is not relevant to the choice of reflections for refinement. *R*-factors based on *F*^2^ are statistically about twice as large as those based on *F*, and *R*- factors based on ALL data will be even larger.

### Fractional atomic coordinates and isotropic or equivalent isotropic displacement parameters (Å^2^)

**Table:**

	*x*	*y*	*z*	*U*_iso_*/*U*_eq_	
C1	0.5826 (4)	0.80378 (13)	0.84675 (11)	0.0506 (5)	
H1A	0.7233	0.8238	0.8463	0.061*	
C2	0.5523 (5)	0.75694 (16)	0.91185 (12)	0.0647 (7)	
H2A	0.4177	0.7331	0.9110	0.078*	
H2B	0.6544	0.7165	0.9137	0.078*	
C3	0.5705 (6)	0.80629 (17)	0.97458 (13)	0.0761 (8)	
H3A	0.5417	0.7754	1.0143	0.091*	
H3B	0.7101	0.8253	0.9784	0.091*	
C4	0.4241 (6)	0.87304 (17)	0.97238 (12)	0.0745 (8)	
H4A	0.4446	0.9049	1.0120	0.089*	
H4B	0.2839	0.8542	0.9732	0.089*	
C5	0.4569 (5)	0.92060 (15)	0.90914 (11)	0.0643 (7)	
H5A	0.5932	0.9431	0.9102	0.077*	
H5B	0.3576	0.9621	0.9081	0.077*	
C6	0.4339 (4)	0.87160 (12)	0.84510 (11)	0.0486 (5)	
H6A	0.2934	0.8515	0.8436	0.058*	
C7	0.3422 (5)	0.98753 (13)	0.78078 (14)	0.0653 (7)	
H7A	0.3672	1.0132	0.7380	0.078*	
H7B	0.3879	1.0216	0.8165	0.078*	
C8	0.1147 (5)	0.97599 (18)	0.78826 (18)	0.0834 (9)	
H8A	0.0553	0.9367	0.7631	0.100*	
C9	−0.0048 (7)	1.0156 (3)	0.8263 (2)	0.1171 (14)	
H9A	0.0483	1.0554	0.8523	0.141*	
H9B	−0.1444	1.0045	0.8279	0.141*	
C10	0.4402 (4)	0.86967 (12)	0.72229 (11)	0.0483 (5)	
H10A	0.2982	0.8509	0.7218	0.058*	
C11	0.4779 (4)	0.91552 (13)	0.65788 (11)	0.0533 (6)	
C12	0.3334 (5)	0.91742 (15)	0.60669 (13)	0.0693 (8)	
H12A	0.2095	0.8915	0.6120	0.083*	
C13	0.3722 (7)	0.95790 (18)	0.54713 (15)	0.0914 (11)	
H13A	0.2743	0.9585	0.5129	0.110*	
C14	0.5517 (7)	0.9965 (2)	0.53880 (15)	0.0981 (12)	
H14A	0.5765	1.0233	0.4990	0.118*	
C15	0.6952 (6)	0.9957 (2)	0.58882 (17)	0.0956 (11)	
H15A	0.8185	1.0219	0.5830	0.115*	
C16	0.6586 (5)	0.95579 (16)	0.64889 (14)	0.0734 (8)	
H16A	0.7567	0.9563	0.6831	0.088*	
C17	0.5854 (4)	0.80049 (12)	0.72363 (11)	0.0494 (5)	
H17A	0.7274	0.8192	0.7231	0.059*	
C18	0.5511 (4)	0.75131 (14)	0.66111 (11)	0.0541 (6)	
C19	0.6996 (5)	0.74603 (15)	0.61113 (13)	0.0677 (7)	
H19A	0.8235	0.7719	0.6165	0.081*	
C20	0.6668 (6)	0.70288 (18)	0.55319 (15)	0.0857 (10)	
H20A	0.7678	0.7002	0.5200	0.103*	
C21	0.4855 (6)	0.66430 (17)	0.54495 (15)	0.0826 (10)	
H21A	0.4637	0.6352	0.5062	0.099*	
C22	0.3358 (6)	0.66848 (16)	0.59373 (14)	0.0763 (8)	
H22A	0.2119	0.6428	0.5878	0.092*	
C23	0.3702 (4)	0.71126 (15)	0.65195 (13)	0.0642 (7)	
H23A	0.2696	0.7129	0.6853	0.077*	
C24	0.6823 (5)	0.68533 (14)	0.78648 (14)	0.0677 (7)	
H24A	0.6575	0.6576	0.7447	0.081*	
H24B	0.6373	0.6528	0.8234	0.081*	
C25	0.9103 (5)	0.6977 (2)	0.79341 (19)	0.0882 (10)	
H25A	0.9648	0.7397	0.7705	0.106*	
C26	1.0331 (7)	0.6584 (3)	0.8261 (2)	0.1236 (16)	
H26A	0.9870	0.6158	0.8499	0.148*	
H26B	1.1716	0.6714	0.8269	0.148*	
N1	0.4696 (3)	0.91737 (10)	0.78298 (9)	0.0510 (4)	
N2	0.5531 (3)	0.75566 (10)	0.78624 (9)	0.0512 (4)	

### Atomic displacement parameters (Å^2^)

**Table:**

	*U*^11^	*U*^22^	*U*^33^	*U*^12^	*U*^13^	*U*^23^
C1	0.0577 (13)	0.0487 (11)	0.0453 (11)	0.0009 (11)	−0.0019 (11)	0.0009 (9)
C2	0.0857 (18)	0.0610 (15)	0.0474 (12)	0.0109 (15)	−0.0022 (13)	0.0088 (11)
C3	0.102 (2)	0.0804 (18)	0.0453 (13)	0.0067 (19)	−0.0094 (15)	0.0070 (13)
C4	0.108 (2)	0.0775 (17)	0.0380 (11)	0.0065 (18)	−0.0012 (14)	−0.0055 (12)
C5	0.0890 (19)	0.0562 (14)	0.0477 (13)	0.0034 (14)	−0.0046 (13)	−0.0067 (11)
C6	0.0582 (13)	0.0467 (11)	0.0409 (11)	0.0014 (11)	−0.0041 (10)	−0.0008 (9)
C7	0.096 (2)	0.0444 (11)	0.0551 (13)	0.0100 (12)	−0.0002 (15)	0.0012 (11)
C8	0.089 (2)	0.0738 (17)	0.088 (2)	0.0259 (17)	−0.0153 (19)	−0.0051 (17)
C9	0.103 (3)	0.118 (3)	0.130 (3)	0.024 (3)	0.021 (3)	−0.012 (3)
C10	0.0579 (13)	0.0453 (11)	0.0419 (11)	−0.0045 (10)	−0.0003 (11)	0.0008 (9)
C11	0.0703 (15)	0.0473 (12)	0.0422 (11)	−0.0025 (12)	−0.0015 (11)	0.0024 (9)
C12	0.088 (2)	0.0602 (15)	0.0600 (15)	−0.0094 (15)	−0.0194 (14)	0.0080 (12)
C13	0.138 (3)	0.0806 (19)	0.0553 (16)	−0.014 (2)	−0.0286 (19)	0.0168 (15)
C14	0.155 (4)	0.085 (2)	0.0537 (16)	−0.026 (3)	−0.002 (2)	0.0203 (16)
C15	0.113 (3)	0.098 (2)	0.075 (2)	−0.038 (2)	0.012 (2)	0.0172 (18)
C16	0.0829 (19)	0.0798 (18)	0.0574 (14)	−0.0230 (16)	−0.0063 (15)	0.0153 (14)
C17	0.0553 (12)	0.0473 (11)	0.0457 (11)	−0.0027 (10)	0.0035 (11)	0.0010 (9)
C18	0.0682 (15)	0.0476 (12)	0.0464 (12)	0.0014 (12)	0.0053 (11)	−0.0016 (10)
C19	0.0761 (18)	0.0638 (15)	0.0630 (15)	−0.0032 (15)	0.0167 (13)	−0.0039 (13)
C20	0.113 (3)	0.085 (2)	0.0590 (16)	−0.006 (2)	0.0291 (18)	−0.0160 (15)
C21	0.119 (3)	0.0755 (19)	0.0527 (15)	0.002 (2)	0.0028 (17)	−0.0179 (14)
C22	0.090 (2)	0.0706 (17)	0.0681 (17)	−0.0135 (16)	−0.0041 (17)	−0.0106 (14)
C23	0.0728 (16)	0.0665 (15)	0.0534 (13)	−0.0082 (14)	0.0096 (13)	−0.0093 (12)
C24	0.097 (2)	0.0477 (12)	0.0581 (14)	0.0146 (13)	0.0004 (16)	0.0027 (11)
C25	0.084 (2)	0.0784 (19)	0.102 (2)	0.0273 (18)	0.0133 (19)	0.0080 (19)
C26	0.107 (3)	0.137 (4)	0.127 (4)	0.027 (3)	−0.026 (3)	0.007 (3)
N1	0.0688 (12)	0.0407 (9)	0.0434 (9)	0.0014 (8)	−0.0006 (10)	0.0024 (8)
N2	0.0653 (11)	0.0411 (9)	0.0474 (10)	0.0034 (8)	0.0014 (10)	0.0016 (8)

### Geometric parameters (Å, °)

**Table:**

C1—N2	1.473 (3)	C12—C13	1.395 (4)
C1—C6	1.528 (3)	C12—H12A	0.9300
C1—C2	1.536 (3)	C13—C14	1.358 (6)
C1—H1A	0.9800	C13—H13A	0.9300
C2—C3	1.514 (4)	C14—C15	1.360 (5)
C2—H2A	0.9700	C14—H14A	0.9300
C2—H2B	0.9700	C15—C16	1.396 (4)
C3—C4	1.505 (4)	C15—H15A	0.9300
C3—H3A	0.9700	C16—H16A	0.9300
C3—H3B	0.9700	C17—N2	1.478 (3)
C4—C5	1.515 (4)	C17—C18	1.520 (3)
C4—H4A	0.9700	C17—H17A	0.9800
C4—H4B	0.9700	C18—C23	1.381 (4)
C5—C6	1.534 (3)	C18—C19	1.385 (4)
C5—H5A	0.9700	C19—C20	1.386 (4)
C5—H5B	0.9700	C19—H19A	0.9300
C6—N1	1.482 (3)	C20—C21	1.368 (5)
C6—H6A	0.9800	C20—H20A	0.9300
C7—N1	1.479 (3)	C21—C22	1.373 (5)
C7—C8	1.501 (5)	C21—H21A	0.9300
C7—H7A	0.9700	C22—C23	1.389 (4)
C7—H7B	0.9700	C22—H22A	0.9300
C8—C9	1.284 (5)	C23—H23A	0.9300
C8—H8A	0.9300	C24—N2	1.487 (3)
C9—H9A	0.9300	C24—C25	1.506 (5)
C9—H9B	0.9300	C24—H24A	0.9700
C10—N1	1.472 (3)	C24—H24B	0.9700
C10—C11	1.523 (3)	C25—C26	1.235 (5)
C10—C17	1.533 (3)	C25—H25A	0.9300
C10—H10A	0.9800	C26—H26A	0.9300
C11—C12	1.381 (4)	C26—H26B	0.9300
C11—C16	1.381 (4)		
			
N2—C1—C6	109.94 (18)	C11—C12—C13	120.4 (3)
N2—C1—C2	111.09 (18)	C11—C12—H12A	119.8
C6—C1—C2	110.4 (2)	C13—C12—H12A	119.8
N2—C1—H1A	108.5	C14—C13—C12	120.5 (3)
C6—C1—H1A	108.5	C14—C13—H13A	119.7
C2—C1—H1A	108.5	C12—C13—H13A	119.7
C3—C2—C1	111.9 (2)	C13—C14—C15	119.9 (3)
C3—C2—H2A	109.2	C13—C14—H14A	120.1
C1—C2—H2A	109.2	C15—C14—H14A	120.1
C3—C2—H2B	109.2	C14—C15—C16	120.3 (3)
C1—C2—H2B	109.2	C14—C15—H15A	119.8
H2A—C2—H2B	107.9	C16—C15—H15A	119.8
C4—C3—C2	111.5 (2)	C11—C16—C15	120.5 (3)
C4—C3—H3A	109.3	C11—C16—H16A	119.7
C2—C3—H3A	109.3	C15—C16—H16A	119.7
C4—C3—H3B	109.3	N2—C17—C18	111.13 (17)
C2—C3—H3B	109.3	N2—C17—C10	110.05 (18)
H3A—C3—H3B	108.0	C18—C17—C10	109.82 (19)
C3—C4—C5	111.0 (2)	N2—C17—H17A	108.6
C3—C4—H4A	109.4	C18—C17—H17A	108.6
C5—C4—H4A	109.4	C10—C17—H17A	108.6
C3—C4—H4B	109.4	C23—C18—C19	117.9 (2)
C5—C4—H4B	109.4	C23—C18—C17	121.1 (2)
H4A—C4—H4B	108.0	C19—C18—C17	120.9 (2)
C4—C5—C6	111.2 (2)	C18—C19—C20	121.2 (3)
C4—C5—H5A	109.4	C18—C19—H19A	119.4
C6—C5—H5A	109.4	C20—C19—H19A	119.4
C4—C5—H5B	109.4	C21—C20—C19	119.9 (3)
C6—C5—H5B	109.4	C21—C20—H20A	120.1
H5A—C5—H5B	108.0	C19—C20—H20A	120.1
N1—C6—C1	109.56 (19)	C20—C21—C22	120.2 (3)
N1—C6—C5	111.58 (19)	C20—C21—H21A	119.9
C1—C6—C5	110.58 (19)	C22—C21—H21A	119.9
N1—C6—H6A	108.3	C21—C22—C23	119.7 (3)
C1—C6—H6A	108.3	C21—C22—H22A	120.2
C5—C6—H6A	108.3	C23—C22—H22A	120.2
N1—C7—C8	116.1 (2)	C18—C23—C22	121.2 (3)
N1—C7—H7A	108.3	C18—C23—H23A	119.4
C8—C7—H7A	108.3	C22—C23—H23A	119.4
N1—C7—H7B	108.3	N2—C24—C25	116.1 (2)
C8—C7—H7B	108.3	N2—C24—H24A	108.3
H7A—C7—H7B	107.4	C25—C24—H24A	108.3
C9—C8—C7	125.7 (4)	N2—C24—H24B	108.3
C9—C8—H8A	117.2	C25—C24—H24B	108.3
C7—C8—H8A	117.2	H24A—C24—H24B	107.4
C8—C9—H9A	120.0	C26—C25—C24	127.2 (4)
C8—C9—H9B	120.0	C26—C25—H25A	116.4
H9A—C9—H9B	120.0	C24—C25—H25A	116.4
N1—C10—C11	111.29 (17)	C25—C26—H26A	120.0
N1—C10—C17	110.49 (18)	C25—C26—H26B	120.0
C11—C10—C17	109.16 (18)	H26A—C26—H26B	120.0
N1—C10—H10A	108.6	C10—N1—C7	111.74 (19)
C11—C10—H10A	108.6	C10—N1—C6	110.48 (16)
C17—C10—H10A	108.6	C7—N1—C6	112.43 (19)
C12—C11—C16	118.3 (2)	C1—N2—C17	111.06 (16)
C12—C11—C10	121.0 (2)	C1—N2—C24	113.17 (19)
C16—C11—C10	120.8 (2)	C17—N2—C24	110.99 (19)
			
C11—C10—C17—C18	−58.0 (2)		
